# Pharmacovigilance Signal Detection of Drug-Induced Hospitalizations and Mortality: A 5-Year Nationwide Study

**DOI:** 10.3390/healthcare13222921

**Published:** 2025-11-14

**Authors:** Jeongah Min, Jeong Eon Lee, Eunah Cho, Jayoung Im, Yeo Jin Choi

**Affiliations:** 1 Department of Regulatory Science, Graduate School, Kyung Hee University, Seoul 02447, Republic of Korea; 2 Institute of Regulatory Innovation through Science (IRIS), Kyung Hee University, Seoul 02447, Republic of Korea; 3 College of Pharmacy and Institute of Integrated Pharmaceutical Sciences, Kyung Hee University, Seoul 02447, Republic of Korea; 4 College of Arts and Science, New York University, New York, NY 10003, USA

**Keywords:** adverse drug events, hospitalizations, death, steroids, anticoagulants, immunosuppressants

## Abstract

**Background/Objectives:** This study aimed to comprehensively characterize the prevalence and patterns of drug-induced hospitalizations and death and to identify predictors strongly associated with drug-induced death. **Methods:** This study analyzed 29,438 serious adverse event (SAE) reports submitted to the Korea Adverse Event Reporting System (KIDS KAERS DB) database between January 2019 and December 2023. Disproportionality analysis was conducted to detect drug–event associations, and multiple logistic regression was performed to identify independent predictors of mortality. **Results:** Mortality accounted for 7.53% (*n* = 2217) and hospitalization for 93.53% (*n* = 27,532). The strong signals for drug-induced death were observed with steroids (ROR 3.81, 95% CI 3.39–4.27), antidotes (ROR 3.65, 95% CI 2.15–6.18), and anticoagulants (ROR 2.01, 95% CI 1.73–2.34). Immunosuppressants (ROR 9.17, 95% CI 4.75–17.70), diuretics (ROR 3.83, 95% CI 1.42–10.31), and antihyperlipidemics (ROR 3.65, 95% CI 1.72–7.69) were strongly associated with hospitalizations. In multivariate regression, men, aging (OR 1.02, 95% CI 1.02–1.03), use of antidotes (OR 11.37, 95% CI 6.59–19.62), steroids (OR 5.78, 95% CI 4.71–7.08), and anticoagulants (OR 3.60, 95% CI 2.90–4.46) were independent predictors of drug-induced mortality. **Conclusions:** This study emphasizes the need for targeted surveillance and risk-mitigation strategies focusing on anticoagulants, steroids and immunosuppressants, particularly among elderly and multimorbid populations.

## 1. Introduction

Adverse drug events (ADEs) represent a major global public health concern, contributing substantially to patient morbidity and mortality [1]. Recent international data indicate that the annual mortality rate attributable to ADEs increased from 2.05 per 100,000 population (95% CI 0.92–3.18) in 2001 to 6.86 (95% CI 5.76–7.95) in 2019, representing more than a threefold rise over two decades [2]. Moreover, VigiBase, the global spontaneous ADE reporting system, contained 36,406,358 ADE reports as of December 2023, marking an 8.68% increase (3,159,452 additional reports) compared with the previous year, implying a continuous global rise in ADEs [3]. Drug-induced hospitalizations account for a considerable proportion of emergency admissions, and severe cases may result in fatal outcomes [4]. Previous study has further reported that ADE-related hospitalizations range from 9.7 to 383 per 100,000 population, whereas mortality rates range from 0.1 to 7.88 per 100,000 [5]. These findings indicate that ADEs are not only frequent occurrences but also clinically significant outcomes, warranting targeted investigation in vulnerable populations, particularly the elderly. Older adults are particularly vulnerable to ADEs due to the increasing complexity of pharmacotherapy associated with multimorbidity, polypharmacy and age-related physiological changes, which collectively amplifies the risk of drug-related hospitalizations and death [6]. With the continued growth of the aging population, the clinical burden of ADE-related hospitalization and mortality is expected to increase further.

Pharmacovigilance, defined as the science and activities related to the detection, assessment, understanding, and prevention of ADEs or other drug-related problems, plays an essential role in ensuring patient safety and optimizing therapeutic outcomes [7]. Among various types of ADEs, those resulting in death, requires hospitalization or the prolongation of existing hospitalizations, persistent or significant disability or incapacity, or birth defect are classified as serious adverse events (SAEs) [8]. The U.S. Food and Drug Administration (FDA) reported that 6.7% of hospitalized patients experience SAEs, with a fatality rate of 0.32%, making drug-related ADEs the 4th leading cause of death, surpassing diabetes, pneumonia, acquired immune deficiency syndrome (AIDS) and pulmonary disease [9]. Despite growing recognition of the clinical and societal burden of ADEs, substantial evidence gaps remain regarding SAEs [10]. In recent years, the utilization of real-world data (RWD) and real-world evidence (RWE) has emerged as an essential approach to bridge the limitations of clinical trials by capturing safety outcomes in real clinical practice. RWD-based pharmacovigilance studies, unlike randomized controlled trials (RCTs), enables identification of rare, delayed, or population-specific adverse events that may not be detected in pre-approval studies [11]. As a regulatory agency, the FDA has released a framework to support RWD use and RWE in regulatory decision-making, thereby reinforcing the legal basis for RWD and its global significance [12]. 1Clinical trials provide important pre-marketing safety data but are limited by relatively small sample sizes, short follow-up periods and narrowly defined patient populations, thereby failing to capture rare yet clinically meaningful events. Therefore, this study was designed to perform signal detection of drug-induced hospitalizations and deaths using a nationwide database. This study aimed to characterize the prevalence and patterns of drug-induced hospitalization and death, and to identify the patient- and treatment-related predictors strongly associated with SAEs.

## 2. Materials and Methods

### 2.1. Study Design and Data Collection

This was a retrospective pharmacovigilance database study employing cross-sectional analyses of spontaneous ADE reports, conducted in accordance with the Strengthening the reporting of observational studies in epidemiology (STROBE) guideline [13]. This study analyzed SAEs reports, primarily drug-induced hospitalizations or death, recorded in the Korean Adverse Event Reporting System database (KIDS KAERS DB) between 1 January 2019, and 31 December 2023. KIDS KAERS DB was constructed by the Korean Institute of Drug Safety and & Risk Managements (KIDS, Ministry of Food and Drug Safety). KIDS KAERS DB collects spontaneous ADE reports from healthcare professionals, the general public, hospitals, community pharmacies, pharmaceutical companies, and regional pharmacovigilance centers [14]. In the KIDS KAERS DB, serious outcomes are coded according to predefined International Council for Harmonisation (ICH) E2D categories, including “hospitalization required” and “prolonged hospitalization” [15]. However, these subcategories are coded under a single outcome variable labeled as “hospitalization”. Therefore, this study included both new hospital admissions and prolonged hospitalizations. All medications reported with drug-induced hospitalization or death and assess as ‘certain’, ‘probable/likely’, and ‘possible; causality criteria according to World Health Organization-Uppsala Monitoring Centre (WHO-UMC) criteria, were included in the analysis [16]. To ensure the accuracy and completeness of submitted reports, KIDS performs in-depth investigations to verify the causality. These investigations include interviewing patients and healthcare professionals, reviewing patients’ medical records, and collecting scientific pharmacovigilance data from pharmaceutical manufacturers [14]. Any ADEs classified as ‘unlikely’, ‘conditional/unclassified”, or ‘assessable/unclassifiable’, and those with masked (MSK-coded) etiologic medications, were excluded from the analysis. MSK codes are assigned to medication products that are marketed by fewer than 2 pharmaceutical companies. Duplicate reports were identified and removed prior to analysis, with only the most recent record retained when multiple versions existed. Recurrent reports referring to the same patient but involving distinct ADEs were treated as separate cases. Reports finalized as invalid, lacking any serious outcomes, or in which the adverse events preceded drug administration were excluded. In addition, reports involving non-medicinal products such as vitamins, dietary supplements, and herbal preparations were also removed. ADEs were coded according to the Medical Dictionary for Regulatory Activity (MedDRA) terminology and were further classified into system organ class (SOC) for the statistical analysis. The prespecified criteria for data extraction included (1) patient demographic information (age and sex), (2) ADE information (etiologic medications, occurrence and resolution dates, causality assessment, and seriousness), (3) reporter information, and (4) medical history and concomitant medications. The protocol of utilizing KIDS KAERS DB was approved by KIDS (Ministry of Food and Drug Safety) (KIDS KAERS DB 2503A0002), and the study was exempted from review by the Kyung Hee University Institutional Review Board (IRB) (KHSIRB-25-185).

### 2.2. Statistical Analysis

Descriptive statistics were used to summarize the demographic and clinical characteristics of patients with SAE. The normality of continuous variables was evaluated using Kolmogorov–Smirnov normality test. As the age did not follow a normal distribution, it was presented as median and interquartile range (IQR). Categorical variables were expressed as frequencies and percentages. Disproportionality analyses were conducted to evaluate the associations between (1) specific drug class and SAE reports related to death and hospitalization, (2) SOC-type and SAE reports related to death and hospitalizations, and (3) sex and SOC categories. Effect sizes were estimated using reporting odds Ratio (ROR) and 95% confidence interval (CI). To ensure the validity and stability of the disproportionality analysis, we restricted inclusion to drug-event pairs with at least 4 reports in both outcome categories: death compared with non-death, and hospitalization compared with non-hospitalizations [17]. This threshold was based on prior methodological validation demonstrating that the reliability and concordance of disproportionality measures become robust when four or more reports constitute a drug-ADE combination [18]. Univariate logistic regression analysis was performed to identify patient- and treatment-specific predictors related to drug-induced death, including variables such as sex, age, hospitalization, and drug class, and predictors associated with significant associations were subsequently entered into multivariate logistic regression model using a forward selection method. Effect size was presented as odds ratios (OR) with corresponding 95% CIs. Sensitivity analyses were performed by excluding chemotherapeutic agents to minimize potential confounding from underlying malignancy-related mortality. In addition, subgroup analyses were conducted according to age (<60 vs. ≥60 years) to explore potential differences in drug-related hospitalization and mortality across age groups, given that studies have reported a substantial increase in polypharmacy prevalence among patients aged 60 years or older [19,20]. Reports with missing age or sex information were excluded from subgroup analyses but retained in overall descriptive and signal-detection analyses to preserve statistical power and minimize selection bias. All statistical analyses were performed using SPSS Statistics 29.0 (IBM Corp., Armonk, NY, USA) and R 4.5.0 (R Foundation for Statistical Computing, Vienna, Austria), and a two-sided *p*-value of <0.05 was considered statistically significant

## 3. Results

### 3.1. Patient Demographic

**Figure 1:**
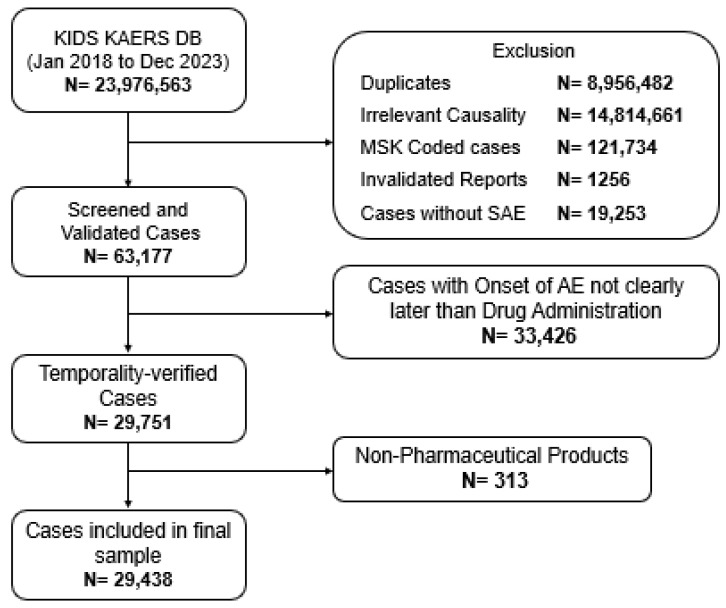
Data acquisition process.

A total of 23,976,563 ADE cases were initially retrieved from the KIDS KAERS DB between January 2018 and December 2023. After excluding duplicates (*n* = 8,956,482), inappropriate causality (*n* = 14,814,661), MSK-coded cases (*n* = 121,734), invalidated reports (*n* = 1256), non-SAE cases (*n* = 19,253) lacking clear temporal relationship (*n* = 33,426), and non-pharmaceutical product cases (*n* = 313), a total of 29,438 drug-induced hospitalization or death cases were included in the analysis (Figure 1). The baseline characteristics of included cases are summarized in Table 1. The median age was 65 years (IQR 21 years). The largest proportions were observed among patients aged 60–69 (27.49%) and 70–79 (23.34%) age groups, while those aged ≥80 years accounted for 9.74% of cases. Men represented 54.02% (*n* = 15,901) of the reports. Drug-induced hospitalization, including new admissions and prolonged hospital stays, comprised 93.53% (*n* = 27,532), whereas death accounted for 7.53% (*n* = 2217). By therapeutic class, anticancer drugs were the most frequently implicated (*n* = 15,196; 51.62%), followed by antibiotics (*n* = 2632; 8.94%), steroids (*n* = 2119; 7.20%), and anticoagulants (*n* = 1566; 5.32%). Other notable contributors included NSAIDs (*n* = 1182; 4.02%) and immunosuppressants (*n* = 1157; 3.93%).

### 3.2. Disproportionality Analysis of Drug-Induced Death or Hospitalizations by Drug Class

The strongest signals for drug-induced death were observed for steroids (ROR 3.81, 95% CI 3.39–4.27), followed by antidotes (ROR 3.65, 95% CI 2.15–6.18), and anticoagulants (ROR 2.01, 95% CI 1.73–2.34) (Table 2). In contrast, markedly lower reporting odds were found for antihyperlipidemic drugs (ROR 0.17, 95% CI 0.07–0.40), diuretics (ROR 0.22, 95% CI 0.08–0.60), analgesics other than nonsteroidal anti-inflammatory drugs (NSAIDs) and opioids (ROR 0.32, 95% CI 0.13–0.77), NSAIDs (ROR 0.51, 95% CI 0.38–0.68), anticonvulsants (ROR 0.60, 95% CI 0.43–0.83), immunosuppressants (ROR 0.69, 95% CI 0.53–0.89), and anticancer drugs (ROR 0.73, 95% CI 0.67–0.80).

For drug-induced hospitalizations, the strongest positive signals were observed for immunosuppressants (ROR 9.17, 95% CI 4.75–17.70), followed by diuretics (ROR 3.83, 95% CI 1.42–10.31), antihyperlipidemic drugs (ROR 3.64, 95% CI 1.72–7.69), antiulcer agents (ROR 3.14, 95% CI 1.40–7.05), other analgesics (ROR 2.68, 95% CI 1.10–6.53), anticoagulants (ROR 1.81, 95% CI 1.39–2.36), anticonvulsants (ROR 1.81, 95% CI 1.26–2.62), and NSAIDs (ROR 1.63, 95% CI 1.22–2.17) (Table 2). On contrary, significantly lower odds of hospitalization were observed for antiviral drugs (ROR 0.41, 95% CI 0.21–0.81), steroids (ROR 0.41, 95% CI 0.36–0.47), antifungals (ROR 0.47, 95% CI 0.23–0.94), and anticancer drugs (ROR 0.70, 95% CI 0.64–0.77).

### 3.3. Disproportionality Analysis of Drug-Induced Death or Hospitalizations by SOC

The results of disproportionality analysis between drug-induced deaths or hospitalizations and SOC-class are summarized in Table 3. The strongest signals related to drug-induced deaths were observed in myo-, endo-, pericardial & valve disorders (ROR 5.52, 95% CI 3.63–8.39), resistance mechanism disorders (ROR 4.66, 95% CI 4.07–5.32), and respiratory system disorders (ROR 4.44, 95% CI 3.99–4.94). On the other hand, the strongest positive signals for drug-induced hospitalizations were observed for metabolic and nutritional disorders (ROR 3.05, 95% CI 1.85–5.01) and psychiatric disorders (ROR 2.80, 95% CI 1.54–5.11). Additional elevated associations were identified for central and peripheral nervous system disorders (ROR 1.88, 95% CI 1.35–2.62), gastrointestinal disorders (ROR 1.73, 95% CI 1.46–2.05), skin and appendage disorders (ROR 1.72, 95% CI 1.42–2.08), and white cell and reticuloendothelial system (RES) disorders (ROR 1.15, 95% CI 1.02–1.31).

### 3.4. Predictors of Drug-Induced Mortality

**Figure 2:**
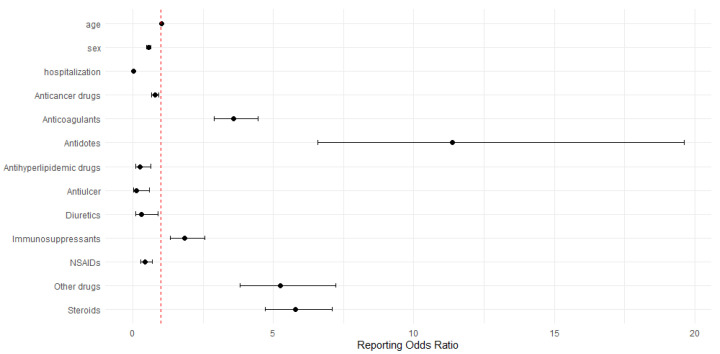
Predictors of drug-induced death risks. The red dotted line indicates a ROR of 1.

Patient- and treatment-specific predictors associated with increased risk of drug-induced mortality included men, aging (OR 1.02, 95% CI 1.02–1.03) and use of antidotes (OR 11.37, 95% CI 6.59–19.62), steroids (OR 5.78, 95% CI 4.71–7.08) or anticoagulants (OR 3.60, 95% CI 2.90–4.46) (Figure 2). In contrast, predictors associated with decreased risk of drug-induced death involved women (OR 0.56, 95% CI 0.49–0.63), previously hospitalized status (OR 0.02, 95% CI 0.02–0.02), and use of antiulcer agents (OR 0.13, 95% CI 0.03–0.60), antihyperlipidemic drugs (OR 0.25, 95% CI 0.09–0.65), diuretics (OR 0.30, 95% CI 0.10–0.91), and NSAIDs (OR 0.44, 95% CI 0.28–0.69).

**Figure 3:**
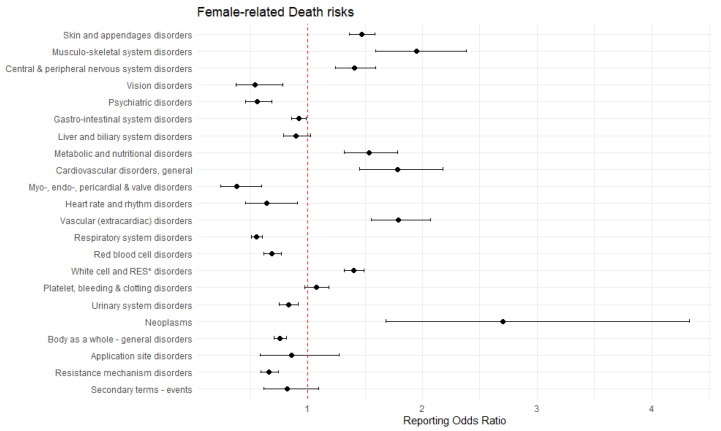
Disproportionality analysis on the association of SOC-based death with women. The red dotted line indicates an ROR of 1.

Among women, the likelihood of reporting death was the highest for neoplasms (ROR 2.70, 95% CI 1.68–4.33), musculoskeletal system disorders (ROR 1.95, 95% CI 1.59–2.38), and vascular (extracardiac) disorders (ROR 1.79, 95% CI 1.55–2.07) (Figure 3). In contrast, reduced reporting odds were observed for myo-, endo-, pericardial & valve disorders (ROR 0.38, 95% CI 0.24–0.60), vision disorders (ROR 0.54, 95% CI 0.37–0.78), and psychiatric disorders (ROR 0.56, 95% CI 0.45–0.68).

### 3.5. Sensitivity Analysis

The highest likelihood of reporting drug-induced death was observed with steroids (ROR 3.79, 95% CI 3.34–4.30), followed by antidotes (ROR 3.14, 95% CI 1.85–5.33) and anticoagulants (ROR 1.77, 95% CI 1.51–2.08) (Table 4). On the other hand, the strongest signal for drug-induced hospitalization was observed with immunosuppressants (ROR 7.82, 95% CI 4.04, 15.13), followed by diuretics (ROR 3.16, 95% CI 1.17–8.51), antihyperlipidemic drugs (ROR 3.01, 95% CI 1.42–6.37), antiulcer drugs (ROR 2.59, 95% CI 1.15–5.83), anticoagulant (ROR 1.51, 95% CI 1.15–1.98) and anticonvulsants (ROR 1.50, 95% CI 1.03–2.17).

The highest likelihood of reporting drug-induced death was observed for body as a whole disorders (ROR 3.26, 95% CI 2.84–3.73), followed by respiratory system disorders (ROR 2.01, 95% CI 1.69–2.37), and platelet, bleeding and clotting disorders (ROR 1.70, 95% CI 1.28–2.27) (Table 5). In contrast, the likelihood of reporting drug-induced hospitalization was the highest for central and peripheral nervous system disorders (ROR 12.73, 95% CI 4.75–34.08), followed by urinary system disorders (ROR 1.84, 95% CI 1.24–2.74) and skin and appendages disorders (ROR 1.30, 95% CI 1.07–1.60).

### 3.6. Subgroup Analysis

In patients aged <60 years, the likelihood of reporting death was highest with anticoagulants (ROR 29.24, 95% CI 22.63–37.78), followed by steroids (ROR 25.61, 95% CI 19.53–33.58) (Table 6). Among patients aged ≥60 years, elevated reporting odds of death were observed with antidotes (ROR 6.29, 95% CI 3.51–11.27), antivirals (ROR 3.15, 95% CI 1.35–7.29), steroids (ROR 2.79, 95% CI 2.36–3.28), metabolic disorder drugs (ROR 1.87, 95% CI 1.18–2.99), opioids (ROR 1.77, 95% CI 1.01–3.13), and immunosuppressants (ROR 1.77, 95% CI 1.01–3.13). Immunosuppressants (ROR 3.76, 95% CI 1.77–7.98) and NSAIDs (ROR 3.42, 95% CI 1.27–9.21) were associated with increased odds of hospitalizations in patients aged <60 years. In contrast, antihyperlidemics (ROR 4.16, 95% CI 1.71–10.09), diuretics (ROR 3.20, 95% CI 1.19–8.64), anticoagulants (ROR 2.40, 95% CI 1.70–3.40), and NSAIDs (ROR 2.27, 95% CI 1.51–3.43) were associated with substantially higher odds of hospitalizations in elderly patients aged 60 years and older.

The reporting odd of drug-induced death in patients aged <60 years was substantially higher with resistance mechanism disorders (ROR 29.41, 95% CI 22.57–38.34), neoplasms (ROR 5.81, 95% CI 2.67–12.65), and metabolic and nutritional disorders (ROR 5.65, 95% CI 3.68–8.69). On the other hand, elderly patients (≥60 years) were more likely to report drug-induced death involving myo-, endo-, pericardial & valve disorders (ROR 6.85, 95% CI 4.39–10.69), respiratory disorders (ROR 3.32, 95% CI 2.92–3.77), urinary system disorders (ROR 1.27, 95% CI 1.03–1.57), and cardiovascular disorders (ROR 1.46, 95% CI 1.05–2.04) (Table 7). On the other hand, the odds of reporting drug-induced hospitalizations were significantly higher with urinary system disorders (ROR 5.35, 95% CI 1.99–14.39) and skin and appendage disorders (ROR 3.02, 95% CI 1.87–4.87) in patients aged <60 years. Elderly patients were more likely to report hospitalizations in red blood cell disorders (ROR 8.33, 95% CI 4.14–16.76), vascular disorders (ROR 5.74, 95% CI 2.56–12.88), respiratory disorders (ROR 3.64, 95% CI 2.65–12.88), metabolic and nutritional disorders (ROR 2.96, 95% CI 1.74–5.05), central and peripheral nervous system disorders (ROR 1.49, 95% CI 1.06–2.09), and gastrointestinal disorders (ROR 1.74, 95% CI 1.42–2.13).

## 4. Discussion

### 4.1. Key Findings and Interpretations

This study comprehensively evaluated SAEs reported to the nationwide pharmacovigilance reporting system, KIDS KAERS DB, between 1 January 2019 and 31 December 2023. The majority of cases occurred in patients aged over 60 years, confirming that the elderly population is particularly vulnerable to drug-related complications. ADE cases involving drug-induced hospitalizations accounted for over 90% of events. Strong signals for drug-induced death were observed with steroids, antidotes, and anticoagulants. In contrast, immunosuppressants, diuretics, and antihyperlipidemic agents were strongly associated with hospitalizations. At the SOC-level, the strongest signals related to drug-induced deaths were observed for myocardial, endocardial, pericardial and valve disorders, resistance mechanism disorders, and respiratory system disorders. Conversely, metabolic and nutritional disorders and psychiatric disorders demonstrated the strongest positive signals for drug-induced hospitalizations. Predictors associated with an increased risk of drug-induced mortality included aging, and use of antidotes, steroids or anticoagulants.

Corticosteroids and immunosuppressants were associated with an increased likelihood of serious drug-related outcomes in this study (Table 2 & Figure 2). The observed associations between corticosteroid use and mortality may reflect both drug-related toxicities and the vulnerability of patients for whom steroids are prescribed [21]. Corticosteroids are typically indicated for patients who have chronic inflammatory or autoimmune diseases, and prolonged exposure to corticosteroids is associated with impaired immune function, subsequently increasing susceptibility to severe infections [21]. Furthermore, long-term corticosteroids can exacerbate underlying comorbidities, including metabolic and cardiovascular disorders, thereby further elevating the risk of hospitalizations and death [22,23]. Importantly, drug-induced mortality signals for corticosteroid-associated mortality remained robust in sensitivity analyses, suggesting that this association was not solely attributable confounding from the underlying malignancy.

In contrast, immunosuppressants demonstrated the strongest association with drug-induced hospitalization, consistent with findings from a Spanish pharmacovigilance study [24]. The increased likelihood of hospitalizations among immunosuppressant users can be explained by both the pharmacological properties of these agents and the characteristics of patient populations in which they are used [25]. Immunosuppressants are commonly prescribed in transplant recipients or patients with severe autoimmune disorders, who require intensive prolonged immunosuppression [26]. This trend has been consistently observed across other large-scaled pharmacovigilance datasets, including FDA Adverse Event Reporting System (FAERS), where immunosuppressant related reports often involve severe infections or systemic complications, resulting in hospitalizations [27]. These patients are consequently more susceptible to opportunistic infections, organ dysfunction, and drug–drug interactions, all of which can collectively contribute to higher hospitalization rates [28]. Moreover, in clinical practice, concomitant use of corticosteroids and immunosuppressants can synergistically amplify immunosuppressive effects, thereby further heightening the risk of severe infection and other complications [29]. Hence, considering the heightened vulnerability to severe infections, metabolic complications, and organ dysfunctions, healthcare professionals should adopt active monitoring strategies, including routine assessment of infection risk, metabolic parameters, and comorbidity status, to mitigate preventable adverse outcomes in these high-risk populations. Furthermore, from a regulatory perspective, these findings highlight the need for targeted formulary-based monitoring programs and safety alerts for patients receiving corticosteroids or immunosuppressants.

Similar to the previous study utilizing VigiBase, which also reported a predominance of anticancer drug-related adverse event reports, anticancer agents accounted for 51.62% of all serious cases in our dataset [30]. However, anticoagulant use had the strongest signals associated with serious drug-related outcomes in this study, and this signal remained consistent in the sensitivity analyses (Table 2 and Table 4 & Figure 2). Subgroup analyses further demonstrated that anticoagulant-related mortality was particularly pronounced in patients aged <60 years, whereas elevated hospitalization risks were observed in older adults (≥60 years) (Table 6). Previous studies have reported that approximately 70% of deaths among anticoagulants users were attributable to cardiovascular disease, while only 6% resulted from stroke or embolism [30,31]. This finding suggests that underlying disease burden plays a major role in mortality risk, implying the need for drug safety management strategies that extend beyond bleeding prevention to include comprehensive comorbidity management. The major adverse effects of anticoagulants are well established and primarily include major bleeding events such as intracranial hemorrhage and gastrointestinal bleeding, which represent the leading causes of anticoagulant-associated morbidity and mortality [32]. Bleeding risk is particularly elevated among older adults due to age-related physiological changes, polypharmacy, and the high prevalence of comorbid cardiovascular disease, all of which collectively increase susceptibility to bleeding [33]. Despite the widespread use of anticoagulants, routine bleeding risk monitoring is not uniformly implemented in clinical practice, with the exception of warfarin, which requires regular international normalized ratio (INR) monitoring. Considering the substantial comorbidity burdens of patients prescribed with anticoagulants, this study indicated the need for careful patient selection, individualized dosing, and regular monitoring of anticoagulant therapy, especially in older adults and patients with multiple comorbidities. Moreover, integration of validated bleeding risk assessment tools, standardized bleeding-risk checklists, and patient education on early signs of bleeding should be prioritized to minimize preventable adverse outcomes.

In this study, most antidote-related cases were attributed to naloxone, which is primarily used for the reversal of opioid overdose (Table 2). The strong mortality signal observed with antidote use likely reflects the underlying clinical circumstances in which naloxone is administered, a life-threatening opioid intoxication [34]. Patients receiving naloxone often present with profound respiratory depression, multi-substance overdose, delayed medical presentation, or comorbid organ dysfunction, all of which contribute to high baseline mortality risk [35]. Importantly, the association between antidote use and mortality persisted even in sensitivity analyses excluding chemotherapeutic agents, suggesting that this signal is not fully explained by confounding from malignancy (Table 4). Naloxone remains the standard of care for opioid overdose reversal, with a well-established safety profile [34]. Its primary mechanism of benefit lies in rapidly reversing opioid-induced respiratory depression, thereby preventing mortality. The rapid reversal of opioid-induced respiratory depression is the primary mechanism by which naloxone reduces mortality. However, naloxone administration can occasionally precipitate acute withdrawal symptoms such as vomiting, tachycardia or agitation, especially in opioid-dependent individuals, which in rare cases may contribute to adverse outcomes [36]. Therefore, mortality signal associated with naloxone use should be interpreted with caution, as it is more likely to serve as a marker of the severity of the underlying overdose rather than a direct drug effect. Hence, recognition of opioid overdose, prompt naloxone administration, and close post-overdose monitoring are critical in clinical practice. Moreover, enhanced pharmacovigilance monitoring and registry-based monitoring of naloxone use may further elucidate patient characteristics and outcomes, providing a clearer understanding of overdose severity and improving strategies to reduce mortality among high-risk populations. Furthermore, regulatory policies such as national opioid safety programs, mandatory naloxone distribution protocols, and integration of opioid overdose monitoring into pharmacovigilance system are warranted to mitigate opioid-related hospitalizations and mortalities.

Aging and sex-related differences were identified as significant predictors of drug-induced mortality in this study (Figure 2 and Figure 3). In the context of aging, previous studies have reported consistent findings. For example, a pharmacovigilance study utilizing VigiBase, reported fatal ADEs were predominantly observed in patients over 75 years of age [30]. Older adults are particularly vulnerable to ADEs due to multimorbidity, polypharmacy and age-related physiological changes, including reduced renal and hepatic clearance, and altered pharmacodynamics [6]. These factors contribute to increased susceptibility to drug toxicity. Our subgroup analyses revealed that elderly patients were more likely to report drug-induced deaths related to cardiovascular, respiratory, and urinary system disorders, which involve vital organ systems essential for survival (Table 6 and Table 7). Age-related decline in cardiac reserve, pulmonary function, and renal capacity, in alignment with underlying comorbidities, may amplify the lethality of drug-induced injuries in older adults [6]. Women were associated with a significantly lower likelihood of mortality compared with men, consistent with findings from previous studies [37,38]. The precise underlying mechanisms of sex differences remain incompletely understood, but several biological and clinical factors have been proposed. Men generally differ in body composition, including lower fat mass but higher muscle mass [39]. Moreover, sex-related variations in cytochrome P450 enzyme activity and drug transporter function may also affect pharmacokinetics of drug, leading to differential toxicity risks [40]. Nevertheless, considering that most patients in this population are elderly, the observed sex differences are likely influenced by complex interactions between biological sex, comorbidity burden, and age-related vulnerability. Therefore, further studies are warranted to perform stratified analyses by age, sex, comorbidities and concomitant medication use to determine population-specific risks. Meanwhile, healthcare professionals should adopt clinical risk assessment and monitoring strategies that incorporate patient age, sex, and comorbidity profiles.

### 4.2. Limitation

This study has several limitations to be acknowledged. First, due to the inherent nature of the voluntary reporting system, reporting bias is likely to be present. Spontaneous reporting depends on the awareness and motivation of healthcare professionals as well as patients, which can lead to both underreporting of less severe cases and overrepresentation of unusual or severe adverse events. Consequently, the database may not fully capture the true incidence or distribution of adverse drug events in the patient populations. Moreover, differences in reporting practices across institutions and over time may have further influenced the observed patterns. Second, the results of this study primarily demonstrate statistical associations rather than causal relationships due to the nature of observational study design. We identified significant association between certain drug classes and mortality or hospitalization; however, definite causal inferences from drug exposure cannot be established. Moreover, several important confounding variables could not be fully addressed due to limited clinical detail in the database. Information on drug dosage, treatment duration, route of administration, and concomitant medications was not consistently available, making it difficult to assess dose–response relationships or drug–drug interactions. Similarly, the severity of underlying comorbidities and the presence of acute illness were not captured, which could have influenced both prescribing decisions and patient outcomes. Fourth, the database did not distinguish between “new hospital admission” and “prolonged hospitalizations” as both were aggregated under a single outcome category of “hospitalization”. Consequently, it was not possible to determine whether drug-related hospitalization represented a new admission event or an extension of an existing hospital stay. Moreover, detailed clinical information such as reason for hospitalization, duration of inpatient care, or outcomes after discharge was not available, which may limit the interpretability of hospitalization related findings. Moreover, the true incidence of ADE could not be calculated as the database lacks exposure denominator. Nevertheless, minimal bias from ADE cases was expected because the Korea Institute of Drug Safety and Risk Management (Ministry of Food and Drug Safety) performs in-depth investigations of ADE reports by collecting scientific pharmacovigilance data from manufacturers, reviewing patients’ medical charts, and consulting with healthcare professionals appointed by the institution. Despite these limitations, this study provides valuable real-world evidence regarding drug-induced hospitalizations and mortality at the nationwide level. This study emphasizes the importance of strengthening pharmacovigilance systems in identifying drug classes and patient subgroups at elevated risk for drug-induced hospitalizations and mortality. Furthermore, routine implementation of clinical monitoring strategies to minimize drug-related risks is crucial for early detection of ADEs, thereby improving patient safety.

## 5. Conclusions

This nationwide drug surveillance study comprehensively analyzed SAEs involving drug-induced hospitalizations and death from 2019 to 2023. Disproportionality analyses highlighted distinct safety signals: steroids, antidotes, and anticoagulants were most strongly associated with drug-induced deaths, whereas immunosuppressants, diuretics, and antihyperlipidemic drugs had strong associations with drug-induced hospitalizations. SOC analysis further revealed strong signals for cardiovascular, respiratory, and resistance mechanism disorders in fatal cases, and for metabolic, psychiatric, and nervous system disorders in hospitalizations. Age-stratified subgroup analyses demonstrated notable differences in drug classes and clinical conditions contributing to severe outcomes, with younger patients more frequently associated with anticoagulants and steroids, whereas elderly patients exhibited higher reporting frequencies with antifungals, diuretics, anticoagulants, and NSAIDs. Multivariate analysis confirmed that men, aging, use of certain drug classes such as antidotes, steroids, and anticoagulants were independent predictors of drug-induced mortality. These results emphasize the importance of strengthening pharmacovigilance systems for early detection of safety signals in high-risk populations, particularly elderly patients and those receiving drug classes such as antidotes, steroids, anticoagulants, and immunosuppressants for early detection of safety signals. Further studies are warranted to perform stratified analyses by age, sex, comorbidities and concomitant medication use to determine population-specific risks. These results emphasize the importance of integrating pharmacovigilance evidence into national regulatory frameworks to enhance proactive risk monitoring strategies to advance pharmacovigilance policy development, prioritizing surveillance efforts toward high-risk drug classes and population through evidence-based risk assessment and targeted safety interventions.

## Figures and Tables

**Table 1:** Baseline demographic characteristics of patients.

Characteristics	No. of Cases (% Relative Frequency) or Median (IQR)
**Age (years) ^a^**	**Median: 65 IQR: 21**
0~9	1448 (4.92%)
10~19	1511 (5.13%)
20~29	492 (1.67%)
30~39	940 (3.19%)
40~49	1846 (6.27%)
50~59	3811 (12.95%)
60~69	8092 (27.49%)
70~79	6872 (23.34%)
80~89	2698 (9.17%)
90~	169 (0.57%)
**Sex ^b^**	
Men	15,901 (54.02%)
Women	13,227 (44.93%)
**Causality**	
Certain	3540 (12.03%)
Probable/Likely	6417 (21.80%)
Possible	19,481 (66.18%)
**Death**	
Death	2217 (7.53%)
Non-death	27,221 (92.47%)
**Hospitalization**	
Hospitalization	27,532 (93.53%)
Non-hospitalization	1906 (6.47%)
**Reporting individuals**	
Doctors	15,727 (53.42%)
Pharmacists	2458 (8.35%)
Other healthcare professionals	10,178 (34.57%)
General Public	642 (2.18%)
Unknown	433 (1.47%)
**Medication class**	
Anesthetics	8 (0.03%)
Antibiotics	2632 (8.94%)
Anticancer drugs	15,196 (51.62%)
Anticholinergics	9 (0.03%)
Anticoagulants	1566 (5.32%)
Anticonvulsants	806 (2.74%)
Antidepressants	55 (0.19%)
Antidiabetic	170 (0.58%)
Antidotes	79 (0.27%)
Antiemetic drugs	16 (0.05%)
Antifungals	70 (0.24%)
Antihistamines	56 (0.19%)
Antihormonal drugs	39 (0.13%)
Antihyperlipidemic drugs	371 (1.26%)
Antiparkinson drugs	10 (0.03%)
Antiprotozoal drugs	73 (0.25%)
Antipsychotics	242 (0.82%)
Antituberculosis drugs	468 (1.59%)
Antiulcer	276 (0.94%)
Antiviral drugs	70 (0.24%)
ARBs	147 (0.50%)
Beta blockers	73 (0.25%)
Calcium channel blockers	94 (0.32%)
Cognitive enhancers	79 (0.27%)
Contrast agents	87 (0.30%)
Diuretics	224 (0.76%)
Genitourinary system drugs	59 (0.20%)
Hormonal therapy drugs	83 (0.28%)
Hypnotics	56 (0.19%)
Immunostimulants	27 (0.09%)
Immunosuppressants	1157 (3.93%)
Metabolic disorder drugs	246 (0.84%)
Muscle relaxants	88 (0.30%)
NSAIDs	1182 (4.02%)
Opioids	147 (0.50%)
Osteoporosis drugs	21 (0.07%)
Other analgesic drugs	198 (0.67%)
Other blood and blood-forming drugs	218 (0.74%)
Other cardiovascular drugs	142 (0.48%)
Other drugs	412 (1.40%)
Other Gastrointestinal drugs	125 (0.42%)
Other neurological drugs	5 (0.02%)
Other psychiatric drugs	62 (0.21%)
Respiratory system drugs	125 (0.42%)
Rheumatoid arthritis and osteoarthritis drugs	21 (0.07%)
Steroids	2119 (7.20%)
Vaccines	29 (0.10%)

^a^ missing in 1559 cases (5.30%). ^b^ missing in 310 cases (1.05%).

**Table 2:** Association of medications class with Drug-induced death or hospitalizations.

Class	Death ROR (95%CI)	*p*-Value	Hospitalization ROR (95%CI)	*p*-Value
Antibiotics	0.96 (0.82–1.12)	0.576	0.99 (0.84–1.16)	0.895
Anticancer drugs	0.73 (0.67–0.80)	<0.001	0.70 (0.64–0.77)	<0.001
Anticoagulants	2.01 (1.73–2.34)	<0.001	1.81 (1.39–2.36)	<0.001
Anticonvulsants	0.60 (0.43–0.83)	0.002	1.81 (1.26–2.62)	0.002
Antidotes	3.65 (2.15–6.18)	<0.001	N/A	N/A
Antifungals	1.82 (0.90–3.66)	0.096	0.47 (0.23–0.94)	0.034
Antihyperlipidemic drugs	0.17 (0.07–0.40)	<0.001	3.64 (1.72–7.69)	<0.001
Antipsychotics	0.64 (0.36–1.14)	0.131	1.80 (0.92–3.51)	0.085
Antituberculosis drugs	1.02 (0.73–1.44)	0.894	1.56 (0.99–2.45)	0.053
Antiulcer drugs	N/A	N/A	3.14 (1.40–7.05)	0.006
Antiviral drugs	1.37 (0.63–2.99)	0.435	0.41 (0.21–0.81)	0.010
Diuretics	0.22 (0.08–0.60)	0.003	3.83 (1.42–10.31)	0.008
Immunosuppressants	0.69 (0.53–0.89)	0.005	9.17 (4.75–17.70)	<0.001
Metabolic disorder drugs	1.21 (0.78–1.88)	0.40	0.74 (0.47–1.16)	0.189
Muscle relaxants	0.74 (0.30–1.82)	0.512	1.15 (0.47–2.84)	0.762
NSAIDs	0.51 (0.38–0.68)	<0.001	1.63 (1.22–2.17)	0.001
Opioids	1.61 (0.97–2.68)	0.066	0.66 (0.38–1.14)	0.135
Other analgesic drugs	0.32 (0.13–0.77)	0.011	2.68 (1.10–6.53)	0.03
Other cardiovascular drugs	0.73 (0.36–1.50)	0.392	0.91 (0.48–1.74)	0.783
Respiratory system drugs	0.62 (0.27–1.41)	0.251	1.01 (0.49–2.08)	0.973
Steroids	3.81 (3.39–4.27)	<0.001	0.41 (0.36–0.47)	<0.001

**Table 3:** Association between System Organ Class (SOC)-based Death caused by Medications.

System Organ Class	Death ROR (95% CI)	*p*-Value	Hospitalization ROR (95% CI)	*p*-Value
Skin and appendages disorders	0.46 (0.38–0.56)	<0.001	1.72 (1.42–2.08)	<0.001
Central & peripheral nervous system disorders	0.57 (0.42–0.77)	<0.001	1.88 (1.35–2.62)	<0.001
Psychiatric disorders	0.33 (0.19–0.59)	<0.001	2.80 (1.54–5.11)	<0.001
Gastro-intestinal system disorders	0.46 (0.39–0.55)	<0.001	1.73 (1.46–2.05)	<0.001
Liver and biliary system disorders	0.44 (0.32–0.62)	<0.001	1.11 (0.85–1.44)	0.448
Metabolic and nutritional disorders	0.56 (0.39–0.80)	0.002	3.05 (1.85–5.01)	<0.001
Cardiovascular disorders, general	1.37 (0.99–1.91)	0.061	0.64 (0.46–0.89)	0.008
Heart rate and rhythm disorders	1.19 (0.67–2.11)	0.546	0.71 (0.40–1.26)	0.244
Vascular (extracardiac) disorders	1.01 (0.78–1.32)	0.919	1.06 (0.79–1.42)	0.684
Respiratory system disorders	4.44 (3.99–4.94)	<0.001	0.76 (0.65–0.88)	<0.001
Red blood cell disorders	0.09 (0.05–0.16)	<0.001	1.27 (1.00–1.60)	0.052
White cell and RES disorders	0.37 (0.32–0.43)	<0.001	1.15 (1.02–1.31)	0.027
Platelet, bleeding & clotting disorders	0.81 (0.67–0.99)	0.036	0.66 (0.56–0.78)	<0.001
Urinary system disorders	1.10 (0.92–1.31)	0.318	0.86 (0.71–1.04)	0.123
Neoplasms	2.58 (1.45–4.59)	0.001	N/A	N/A
Body as a whole—general disorders	1.58 (1.40–1.78)	<0.001	0.50 (0.44–0.57)	<0.001
Resistance mechanism disorders	4.66 (4.07–5.32)	<0.001	0.57 (0.48–0.69)	<0.001
Secondary terms—events	0.26 (0.10–0.70)	0.008	0.25 (0.18–0.36)	<0.001

Abbreviation: RES: reticuloendothelial system.

**Table 4:** Sensitivity analysis on drug-induced death and hospitalization.

Class	Death Reports (*n* = 1234)	Death ROR (95% CI)	*p*-Value	Hospitalization Reports (*n* = 13,477)	Hospitalization ROR (95% CI)	*p*-Value
Antibiotics	191	0.79 (0.68–0.93)	0.005	2460	0.77 (0.65–0.92)	0.003
Anticoagulants	211	1.77 (1.51–2.08)	<0.001	1507	1.51 (1.15–1.98)	0.003
Anticonvulsants	38	0.51 (0.36–0.71)	<0.001	776	1.50 (1.03–2.17)	0.034
Antidotes	18	3.14 (1.85–5.33)	<0.001	79	N/A	N/A
Antifungals	9	1.56 (0.77–3.15)	0.215	61	0.38 (0.19–0.77)	0.007
Antihyperlipidemic drugs	5	0.14 (0.06–0.34)	<0.001	**7**	3.01 (1.42–6.37)	0.004
Antipsychotics	12	0.55 (0.30–0.98)	0.042	9	1.48 (0.76–2.89)	0.253
Antituberculosis drugs	36	0.88 (0.62–1.24)	0.447	20	1.28 (0.81–2.02)	0.285
Antiulcer drugs	2	N/A	N/A	6	2.59 (1.15–5.83)	0.022
Antiviral drugs	7	1.17 (0.54–2.57)	0.691	10	0.34 (0.17–0.66)	0.002
Diuretics	4	0.19 (0.07–0.51)	<0.001	4	3.16 (1.17–8.51)	0.023
Immunosuppressants	62	0.58 (0.44–0.75)	<0.001	9	7.82 (4.04–15.13)	<0.001
Metabolic disorder drugs	22	1.04 (0.67–1.61)	0.876	21	0.60 (0.38–0.95)	0.028
Muscle relaxants	5	0.63 (0.26–1.57)	0.323	5	0.94 (0.38–2.33)	0.897
NSAIDs	48	0.42 (0.32–0.57)	<0.001	49	1.34 (1.00–1.80)	0.052
Opioids	17	1.38 (0.83–2.30)	0.211	14	0.54 (0.31–0.93)	0.027
Other analgesic drugs	5	0.27 (0.11–0.66)	0.004	5	2.21 (0.91–5.38)	0.081
Other cardiovascular drugs	8	0.63 (0.31–1.28)	0.201	10	0.75 (0.39–1.43)	0.376
Respiratory system drugs	6	0.53 (0.23–1.20)	0.251	8	0.83 (0.40–1.70)	0.609
Steroids	443	3.79 (3.34–4.30)	<0.001	282	0.27 (0.23–0.32)	<0.001

**Table 5:** Association between System Organ Class (SOC)-based drug induced death and hospitalization.

System Organ Class	Death Reports (*n* = 1234)	Death ROR (95% CI)	*p*-Value	Hospitalization Reports (*n* = 13,477)	Hospitalization ROR (95% CI)	*p*-Value
Skin and appendages disorders	110	0.40 (0.32–0.48)	<0.001	2569	1.30 (1.07–1.60)	0.01
Central & peripheral nervous system disorders	5	0.06 (0.02–0.14)	<0.001	845	12.73 (4.75–34.08)	<0.001
Psychiatric disorders	11	0.66 (0.36–1.22)	0.189	175	0.99 (0.52–1.89)	0.984
Gastro-intestinal system disorders	101	0.69 (0.56–0.85)	<0.001	1496	0.84 (0.68–1.04)	0.117
Liver and biliary system disorders	28	0.50 (0.34–0.73)	<0.001	585	1.46 (0.96–2.24)	0.078
Metabolic and nutritional disorders	28	0.54 (0.37–0.79)	0.002	554	2.69 (1.51–4.79)	<0.001
Cardiovascular disorders, general	4	0.20 (0.07–0.54)	0.001	206	1.68 (0.79–3.58)	0.179
Heart rate and rhythm disorders	12	1.09 (0.60–1.98)	0.774	116	0.55 (0.30–0.99)	0.047
Vascular (extracardiac) disorders	59	0.96 (0.73–1.26)	0.748	47	0.79 (0.58–1.07)	0.126
Respiratory system disorders	186	2.01 (1.69–2.37)	<0.001	1155	0.71 (0.57–0.90)	0.004
Red blood cell disorders	11	0.21 (0.12–0.38)	<0.001	548	N/A	N/A
White cell and RES disorders	25	0.42 (0.28–0.63)	<0.001	607	1.40 (0.93–2.10)	0.108
Platelet, bleeding & clotting disorders	56	1.70 (1.28–2.27)	<0.001	389	1.11 (0.70–1.75)	0.661
Urinary system disorders	47	0.61 (0.45–0.82)	0.001	820	1.84 (1.24–2.74)	0.003
Neoplasms	8	1.60 (0.76–3.36)	0.22	61	N/A	N/A
Body as a whole—general disorders	338	3.26 (2.84–3.73)	<0.001	1473	0.31 (0.26–0.37)	<0.001
Resistance mechanism disorders	201	8.1 (6.71–9.79)	<0.001	453	0.47 (0.35–0.63)	<0.001

Abbreviation: RES: reticuloendothelial system.

**Table 6:** Subgroup analysis on likelihood off reporting drug-induced death or hospitalization per drug class.

Class	Age < 60 Death Reports (*n* = 409)	Age < 60 Death ROR (95%CI) (*n* = 409)	*p*-Value	Age ≥ 60 Death Reports (*n* = 1521)	Age ≥ 60 Death ROR (95%CI) (*n* = 1521)	*p*-Value	Age < 60 Hospitalization Reports (*n* = 9727)	Age < 60 Hospitalization ROR (95%CI) (*n* = 9727)	*p*-Value	Age ≥ 60 Hospitalization Reports (*n* = 16,597)	Age ≥ 60 Hospitalization ROR (95%CI)	*p*-Value
Antibiotics	51	1.07 (0.79–1.44)	0.67	140	1.19 (0.99–1.43)	0.066	1134	0.68 (0.5–0.93)	0.014	1303	0.79 (0.65–0.96)	0.019
Anticancer drugs	56	0.16 (0.12–0.21)	<0.001	831	0.94 (0.84–1.04)	0.21	4652	0.50 (0.04–0.63)	<0.001	9273	0.84 (0.75–0.95)	0.004
Anticoagulants	138	29.24 (22.63–37.78)	<0.001	48	0.48 (0.36–0.64)	<0.001	300	N/A	N/A	1057	2.40 (1.70–3.40)	<0.001
Anticonvulsants	0	N/A	N/A	38	1.23 (0.88–1.73)	0.227	425	N/A	N/A	343	0.94 (0.64–1.40)	0.773
Antidotes	0	N/A	N/A	18	6.29 (3.51–11.27)	<0.001	30	N/A	N/A	49	N/A	N/A
Antifungals	**8**	5.47 (2.52–11.88)	<0.001	1	N/A	N/A	35	0.14 (0.07–0.31)	<0.001	24	N/A	N/A
Antihyperlipidemic drugs	0	N/A	N/A	5	0.19 (0.08–0.47)	<0.001	87	N/A	N/A	276	4.16 (1.71–10.09)	<0.001
Antipsychotics	12	1.52 (0.84–2.75)	0.166	0	N/A	N/A	191	0.69 (0.35–1.37)	0.292	42	N/A	N/A
Antituberculosis drugs	0	N/A	N/A	36	1.46 (1.02–2.07)	0.036	165	N/A	N/A	283	1.05 (0.67–1.66)	0.825
Antiulcer	0	N/A	N/A	2	N/A	N/A	199	N/A	N/A	71	0.88 (0.38–2.03)	0.763
Antiviral drugs	0	N/A	N/A	**7**	3.14 (1.35–7.29)	0.008	30	N/A	N/A	23	0.21 (0.10–0.48)	<0.001
Diuretics	0	N/A	N/A	4	0.25 (0.09–0.67)	0.006	44	N/A	N/A	171	3.20 (1.19–8.64)	0.022
Immunosuppressants	12	0.36 (0.20–0.64)	<0.001	**39**	1.40 (1.00–1.96)	0.03	752	3.76 (1.77–7.98)	<0.001	339	N/A	N/A
Metabolic disorder drugs	1	N/A	N/A	21	1.87 (1.18–2.99)	0.008	94	N/A	N/A	123	0.48 (0.29–0.78)	0.003
Muscle relaxants	0	N/A	N/A	5	1.01 (0.40–2.54)	0.98	30	N/A	N/A	53	0.79 (0.31–1.97)	0.610
NSAIDs	2	N/A	N/A	25	0.36 (0.24–0.55)	<0.001	402	3.42 (1.27–9.21)	0.015	716	2.27 (1.51–3.43)	<0.001
Opioids	3	N/A	N/A	14	1.77 (1.01–3.13)	0.048	48	N/A	N/A	85	0.45 (0.25–0.79)	0.006
Other analgesic drugs	0	N/A	N/A	5	0.56 (0.23–1.37)	0.203	87	N/A	N/A	96	1.43 (0.58–3.52)	0.436
Other cardiovascular drugs	0	N/A	N/A	8	0.88 (0.43–1.82)	0.738	37	N/A	N/A	95	0.71 (0.37–1.36)	0.294
Respiratory system drugs	0	N/A	N/A	6	0.95 (0.41–2.18)	0.896	46	N/A	N/A	68	0.84 (0.37–1.94)	0.687
Steroids	115	25.61 (19.53–33.58)	<0.001	200	2.79 (2.36–3.28)	<0.001	256	2.14 (0.79–5.79)	0.133	873	0.35 (0.30–0.42)	<0.001

**Table 7:** Subgroup analysis on the association between System Organ Class (SOC)-based Death caused by Medications.

System Organ Class	Age < 60 Death Reports (*n* = 409)	Age < 60 Death ROR (95% CI)	*p*-Value	Age < 60 Hospitalization Reports (*n* = 9727)	Age < 60 Hospitalization ROR (95% CI)	*p*-Value	Age ≥ 60 Death Reports (*n* = 1521)	Age ≥ 60 Death ROR (95% CI)	*p*-Value	Age ≥ 60 Hospitalization Reports (*n* = 16,597)	Age ≥ 60 Hospitalization ROR (95% CI)	*p*-Value
Skin and appendages disorders	19	0.27 (0.17–0.43)	<0.001	1478	3.02 (1.87–4.87)	<0.001	91	0.75 (0.60–0.93)	0.008	1277	0.97 (0.78–1.20)	0.753
Central & peripheral nervous system disorders	2	N/A	N/A	211	N/A	N/A	44	0.66 (0.49–0.90)	0.009	711	1.49 (1.06–2.09)	0.022
Psychiatric disorders	6	0.82 (0.36–1.86)	0.634	173	1.14 (0.47–2.80)	0.768	6	0.24 (0.11–0.55)	<0.001	260	3.26 (1.45–7.33)	0.004
Gastro-intestinal system disorders	32	0.68 (0.47–0.98)	0.039	1057	0.77 (0.56–1.06)	0.110	113	0.47 (0.39–0.58)	<0.001	2371	1.74 (1.42–2.13)	<0.001
Liver and biliary system disorders	18	1.03 (0.64–1.68)	0.893	399	0.42 (0.28–0.61)	<0.001	18	0.34 (0.21–0.54)	<0.001	550	1.38 (0.95–2.00)	0.093
Metabolic and nutritional disorders	27	5.65 (3.68–8.69)	<0.001	144	N/A	N/A	4	0.08 (0.03–0.20)	<0.001	545	2.96 (1.74–5.05)	<0.001
Cardiovascular disorders, general	0	N/A	N/A	45	N/A	N/A	40	1.46 (1.05–2.04)	0.026	300	0.61 (0.43–0.87)	0.006
Myo-, endo-, pericardial & valve disorders	0	N/A	N/A	21	N/A	N/A	32	6.85 (4.39–10.69)	<0.001	81	N/A	N/A
Heart rate and rhythm disorders	0	N/A	N/A	51	N/A	N/A	10	1.54 (0.79–2.99)	0.026	70	0.52 (0.27–1.01)	0.053
Vascular (extracardiac) disorders	0	N/A	N/A	263	N/A	N/A	16	0.38 (0.23–0.63)	<0.001	453	5.74 (2.56–12.88)	<0.001
Respiratory system disorders	18	1.9 (1.16–3.10)	0.010	245	N/A	N/A	383	3.32 (2.92–3.77)	<0.001	1843	3.64 (2.65–12.88)	<0.001
Red blood cell disorders	0	N/A	N/A	456	N/A	N/A	11	0.13 (0.07–0.24)	<0.001	856	8.33 (4.14–16.76)	<0.001
White cell and RES disorders	8	0.07 (0.04–0.15)	<0.001	2005	0.65 (0.51–0.83)	<0.001	164	0.58 (0.49–0.69)	<0.001	2765	0.98 (0.84–1.14)	0.745
Platelet, bleeding & clotting disorders	34	0.81 (0.67–0.99)	0.879	790	1.05 (0.69–1.58)	0.829	77	0.93 (0.73–1.18)	0.562	820	0.41 (0.34–0.49)	<0.001
Urinary system disorders	31	1.26 (0.87–1.84)	0.224	615	5.35 (1.99–14.39)	<0.001	105	1.27 (1.03–1.57)	0.024	884	0.53 (0.43–0.64)	<0.001
Neoplasms	8	5.81 (2.67–12.65)	<0.001	41	N/A	N/A	0	N/A	N/A	33	N/A	N/A
Body as a whole—general disorders	76	1.70 (1.31–2.20)	<0.001	1185	1.21 (0.84–1.75)	0.305	273	1.93 (1.68–2.23)	<0.001	1604	0.30 (0.26–0.34)	<0.001
Application site disorders	0	N/A	N/A	59	N/A	N/A	0	N/A	N/A	33	N/A	N/A
Resistance mechanism disorders	128	29.41 (22.57–38.34)	<0.001	257	0.46 (0.28–0.75)	0.002	131	2.20 (1.81–2.68)	<0.001	758	1.36 (0.99–1.86)	0.058
Secondary terms—events	1	N/A	N/A	67	0.05 (0.03–0.08)	<0.001	0	N/A	N/A	71	N/A	N/A

Abbreviation: RES: reticuloendothelial system.

## Data Availability

The data presented in this study are available on request from the corresponding author and KIDS due to the institutional policy and ethical concerns.

## Abbreviations

The following abbreviations are used in this manuscript:

ADE	Adverse drug events
CI	Confidence interval
NSAIDs	Nonsteroidal anti-inflammatory drugs
OR	Odds ratio
ROR	Reporting odds ratio
SAE	Serious adverse events
SOC	System Organ Class
WHO-UMC	World Health Organization-Uppsala Monitoring Centre
